# Users and non-users of web-based health advice service among Finnish university students – chronic conditions and self-reported health status (a cross-sectional study)

**DOI:** 10.1186/1472-6947-8-8

**Published:** 2008-01-31

**Authors:** Johanna Castrén, Teppo Huttunen, Kristina Kunttu

**Affiliations:** 1 Medical School, Department of General Practice, 33014 University of Tampere, Finland; 2 Finnish Student Health Service, Töölönkatu 37, 00260 Helsinki, Finland; 3 Pirkanmaa Hospital District, Department of General Practice, 33521 Tampere, Finland; 4 Oy 4Pharma Ltd, Turku, Finland

## Abstract

**Background:**

The Internet is increasingly used by citizens as source of health information. Young, highly educated adults use the Internet frequently to search for health-related information. Our study explores whether reported chronic conditions or self-reported health status differed among Finnish university students using the Finnish Student Health Services web-based health advice service compared with those not using the service.

**Methods:**

Cross-sectional study performed by a national postal survey in 2004. Material: A random sample (n = 5 030) of a population of 101 805 undergraduate Finnish university students aged 19–35. The response rate: 63% (n = 3 153). Main outcome measures: Proportion of university students reporting use a of web-based health advice service, diagnosed chronic conditions, and self-reported health status of users and non-users of a web-based health advice service. Statistical methods: Data were presented with frequency distributions and cross-tabulations and the χ^2 ^test was used.

**Results:**

12% (n = 370) of Finnish undergraduate students had used the web-based health advice service and were identified as 'users'. The proportion of male students reporting allergic rhinitis or conjunctivitis was greater among users than non-users (24%, n = 22 vs. 15%, n = 154, χ^2^, P = .03). The proportion of female students reporting chronic mental health problems was greater among users than non-users (12%, n = 34 vs. 8%, n = 140, χ^2^, P = .03). There was no statistical significance between the group differences of male or female users and non-users in self-reported health status (good or fairly good, average, rather poor or poor).

**Conclusion:**

Among young, highly educated adults the use of a web-based health advice service is not associated with self-reported health status. However, a web-based health advice service could offer support for managing several specific chronic conditions. More research data is needed to evaluate the role of web-based health advice services that supplement traditional forms of health services.

## Background

The Internet is increasingly used by citizens as source of health information. Young adults are active users of the Internet; they also use it frequently to search for health-related information [1-9]. There are great expectations and high hopes for eHealth services. They are considered to be a significant factor in maintaining patient satisfaction and patient empowerment, and they are expected to help increase the efficiency of health care [10-13].

The Finnish Student Health Service (FSHS) provides primary health care services for approximately 140 500 Finnish university students. Over 80% of funding for the FSHS comes from the public sector. Students pay an annual health care fee of 35 euros as a part of their compulsory student union membership fee. FSHS services include preventive health care, health care and medical treatment provided by a GP and the most relevant specialist services for the age group, mental health care and oral health care.

Since 2002, the services have also included a web-based health advice service through which health care professionals (general practitioners, nurses and oral health care professionals) give instructions and advice on health and illnesses to anonymous students. The service is a text-based consultation service (ask-the-doctor service) provided trough the FSHS website. It is free of charge and provides a question-answer service on the following topics: sexual health, asthma and allergies, travel medicine and vaccinations, oral health, and mental health. A question-answer database that has been compiled on unselected health topics is also available for use with search words. The users of the service cannot be recognized, nor can the use of the service be linked to a student's electronic patient records. In 2006, the service was asked a total of 2 770 questions. All the questions were answered.

There is wide spread opinion, that standards and ethical guidelines regarding electronic communication between a patient and a doctor without personal contact are incomplete. Both patients and health professionals must be aware of this and respect the major limitations of this type of communication. It is unethical to provide medical care without adequate information on a patient's medical history [13-17]. Recent studies by Umefjord from Sweden do, however, provide evidence that proper, anonymous web-based health advice services are being well received by patients and doctors [15,18-20]. There is evidence that patients with one or more chronic condition actively use the Internet to search for health-related information [3,21-24]. Of the chronic conditions stigmatized conditions such as depression and anxiety seems to be linked with the active use of the Internet generally as source of health-related information as well as with the active use of Internet-based communication services with health professionals [3,23-26].

There is also evidence of differences in the health status of citizens who use traditional 'offline' sources of health information and citizens who use 'online' information from the Internet for the same purpose. 'Online' seekers are more likely to be healthier and happier than those seeking health information elsewhere. This supports the 'digital divide' identified as one of the major risks of eHealth [12,27].

Our previous study identified some major general background factors (female gender, in habiting one of the larger cities) that were linked with use of the FSHS web-based health advice service [9]. In this study we focused on the health-related factors linked with the use of web-based health advice services. We examined whether reported chronic conditions or self-reported health status differed among students using the FSHS web-based health advice service compared with those not using the service.

## Methods

### Study design

Our explorative study was carried out in a form of a cross-sectional study as a part of the 'Student Health Survey 2004', a national survey among Finnish undergraduate university students aged 19–35 as. The study population in the 'Student Health Survey 2004' was 101 805. A random sample stratified by district area was drawn. The sample size was 5 030 students of whom 46% (n = 2 300) were men and 54% (n = 2 730) women. Data was collected by means of a postal questionnaire, with three repeat questionnaires. All students gave their informed consent to participate in the study by answering the questionnaire. The 'Student Health Survey 2004' was approved by the medical ethics committee of the Hospital District of Southwest Finland.

Overall 3 153 out of 5 030 students returned a completed or an almost completed survey giving a response rate of 63%; for men 49% (n = 1 132) and for women 74% (n = 2 021). The respondents did not significantly differ from the total study population with respect to gender, age, university, and field of study (Table 1, 2, and 3) [28].

**Table 1:** The Student Health Survey 2004: Study population and respondents by gender

	Study population*		Respondents	
	(n = 101 805)		(n = 3 153)	

	n	%	n	%

Gender				
Men	46 388	46	1 132	36
Women	55 417	54	2 021	64

*Source: Finnish Student Health Service 2003

**Table 2:** Undergraduate students in Finland* and the respondents of the 'Student Health Survey 2004' by age

	Undergraduate students		Respondents	
	(n = 123 544)		(n = 3 153)	

	n	%	n	%

Age group				
Under 22	28 280	23	671	21
22–24	38 917	32	1 090	35
25–29	41 874	34	1 083	34
30–34	14 473	12	309	10

*Source: Education Statistics 2003. Statistics Finland

**Table 3:** Undergraduate students in Finland who are entitled to health care services provided by the Finnish Student Health Service* and the respondents of the 'Student Health Survey 2004' by university city

	Undergraduate students		Respondents	
	(n = 138 544)		(n = 3 153)	

	n	%	n	%

University city				
Helsinki Metropolitan Area	50 595	37	1 091	35
Tampere	20 196	15	439	14
Turku	18 863	14	429	14
Jyväskylä	11 971	9	316	10
Oulu	12 430	9	296	9
Other^†^	24 049	17	569	18
Missing data	0	0	13	0.4

*Source: Finnish Student Health Service 2003

^†^Small university cities

### Study variables

The 'Student Health Survey 2004' questionnaire included a total of 122 questions related to physical, mental, and social health, health behaviour, health-related attitudes, and the use of health services. The survey also included four special questions related to the theme eHealth [28].

The reported use of the FSHS web-based health advice service was chosen as our primary independent variable. Concerning use of web-based health advice service following question was asked of students: 'Have you used the FSHS web-based health advice service?' If they answered yes, then they were categorised as 'users'. If they answered no they were categorized as 'non-users'.

Two groups were chosen for comparison; all those who had used the service at least once were classified as users. The idea of the rough classification chosen was to include students who had used the service once to represent 'normal' web-based health advice service users in the study data. According to our earlier research, the highest average value for use of web-based health advice service was 1.9, the median was 1.0 and the range of use during the previous year was 1–10 times [9].

Diagnosed chronic conditions and self-reported health status were chosen as the dependent variables in order to examine whether health-related variables differed among students using the FSHS web-based health advice service compared with those not using the service. Concerning chronic conditions following question was asked: 'Do you have any chronic disease, illness or disability diagnosed by a physician, dentist, or psychologist which has produced symptoms or required treatment during the last 12 months?'

Concerning health status a five-stepped rating scale of individuals' own health was chosen. The given alternatives for self-reported health status were: 'good', 'fairly good', 'average', 'rather poor' or 'poor'.

### Statistical methods

Data were presented with frequency distributions and cross-tabulations. The variables were tested using the χ^2 ^test. The tests were made two-sided and P-values below .05 were regarded as statistically significant. Statistical analyses were performed using SAS^® ^version 8.2 (SAS Institute Inc., Cary, NC, USA).

## Results

Out of a total of 3 153 respondents 3 114 individuals answered the question 'Have you used the FSHS web-based health advice service?' Of these students 36% (n = 1 113) were men and 64% (n = 2 001) women (Table 4).

**Table 4:** Distribution of men and women in the study population, in the random sample, among the respondents, among the students who responded to the question concerning use of the web-based health advice service, and among the users of the web-based health advice service in the 'Student Health Survey 2004'

	Men		Women		All	
Study Population	46 388	46%	55 417	54%	101 805	100%
Random sample	2 300	46%	2 730	54%	5 030	100%
Study respondents	1 132	36%	2 021	64%	3 153	100%
Students who responded to the question concerning use of the web-based health advice service	1 113	36%	2 001	64%	3 114	100%
Users of the web-based health advice service	92	25%	278	75%	370	100%

In 2004, 12% (n = 370) of Finnish undergraduate students had used the web-based health advice service provided by the Finnish Student Health Service. The majority (75%, n = 278) of these students – who are further referred to as 'users' – were women (Table 4).

The majority (72%, n = 2 206) of Finnish undergraduate students in the 'Student Health Survey 2004' reported having one or more chronic condition. The most common chronic conditions for both sex were refractive errors (29%, n = 883), dental caries (28%, n = 871), and allergic rhinitis or conjunctivitis (17%, n = 530) (Table 5).

**Table 5:** The most common reported separate chronic conditions among Finnish undergraduate students in the 'Student Health Survey 2004'

	Undergraduate Students	
	(n = 3 059–3 065)	

Chronic Condition*		
Refractive Errors or other Eye Disorders	883	29%
Dental caries	871	28%
Allergic Rhinitis, or Conjunctivitis	530	17%
Atopic Dermatitis	302	10%
Infected Wisdom teeth	218	7%
Migraine	196	6%
Lactose Intolerance	192	6%
Depression	153	5%
Musculoskeletal and Connective Tissue Disorders	150	5%
Asthma or other Pulmonary Disorders	146	5%

* Reported chronic diseases, illnesses or disabilities diagnosed by a physician, dentist, or psychologist, which had produced symptoms or required treatment during the last 12 months

Compared with male non-users, male users had more frequently been diagnosed for allergic rhinitis or conjunctivitis (24%, n = 22 vs. 15%, n = 154, χ^2^, P = .03). Compared with female non-users, female users had more frequently been diagnosed for psychiatric disorders (12%, n = 34 vs. 8%, n = 140, χ^2^, P = .03), kidney and urinary tract disorders (7%, n = 18, vs 4%, n = 66, χ^2^, P = .04), and cardiovascular disorders (6%, n = 17 vs. 3%, n = 42, χ^2^, P = .001) (Table 6).

**Table 6:** Numbers and proportions of Finnish undergraduate students in the 'Student Health Survey 2004' diagnosed with chronic conditions in the non-user group and in the user group by gender, as well as the statistical significance between the proportions of chronic conditions in the non-user and in the user groups

	Men				
	Non-users		Users		P*

	(n = 996–999)		(n = 91–92)		

Chronic Condition^†^					
One or more diagnosed chronic conditon of any type	634	64%	67	73%	0.07
Dental Caries or other Oral Disorders	283	28%	29	32%	0.52
Refractive Errors or other Eye Disorders	250	25%	24	26%	0.83
Allergic Rhinitis, or Conjunctivitis	154	15%	22	24%	***0.03***
Atopic Dermatitis	65	7%	10	11%	0.12
Psychiatric Disorders (excl. Eating Disorders)	51	5%	7	8%	0.31
Acne or other Dermatological Disorders (excl. Atopic Dermatitis)	50	5%	8	9%	0.13
Ear, Nose and Throat Disorders	50	5%	4	4%	0.78
Musculoskeletal and Connective Tissue Disorders	44	4%	6	7%	0.34
Lactose Intolerance	43	4%	7	8%	0.15
Asthma or other Pulmonary Disorders	37	4%	3	3%	0.83
Migraine	37	4%	1	1%	0.19
Cardiovascular Disorders	26	3%	3	3%	0.71
Gastrointestinal Disorders (excl. Lactose Intolerance)	24	2%	4	4%	0.26
Disorders of Male Reproductive Organs	12	1%	2	2%	0.43
Diabetes	9	1%	0	0%	0.36
Kidney and Urinary Tract Disorders	4	0.4%	0	0.0%	0.54
Thyroid Disorders	2	0.2%	0	0.0%	0.67
Eating Disorders	1	0.1%	0	0.0%	0.76
Epilepsia or other Neurological Disorders (exlc. Migraine)	0	0%	0	0%	xx

	Women				

	Non-users		Users		P*

	(n = 1 697–1 699)		(n = 275)		

One or more diagnosed chronic conditon of any type	1 297	76%	217	79%	0.35
Dental Caries or other Oral Disorders	572	34%	87	32%	0.50
Refractive Errors or other Eye Disorders	534	31%	85	31%	0.86
Allergic Rhinitis, or Conjunctivitis	297	18%	54	20%	0.39
Atopic Dermatitis	189	11%	35	13%	0.44
Psychiatric Disorders (excl. Eating Disorders)	140	8%	34	12%	***0.03***
Acne or other Dermatological Disorders (excl. Atopic Dermatitis)	137	8%	29	11%	0.17
Ear, Nose and Throat Disorders	137	8%	28	10%	0.24
Musculoskeletal and Connective Tissue Disorders	85	5%	19	7%	0.19
Lactose Intolerance	122	7%	18	7%	0.70
Asthma or other Pulmonary Disorders	88	5%	17	6%	0.49
Migraine	135	8%	23	8%	0.81
Cardiovascular Disorders	42	3%	17	6%	***0.001***
Gastrointestinal Disorders (excl. Lactose Intolerance)	87	5%	15	6%	0.82
Diabetes	9	1%	3	1%	0.27
Kidney and Urinary Tract Disorders	66	4%	18	7%	***0.04***
Thyroid Disorders	23	1%	6	2%	0.29
Eating Disorders	26	2%	4	2%	0.92
Epilepsia or other Neurological Disorders (exlc. Migraine)	6	0.4%	2	1%	0.37
Gynaecological Disorders	134	8%	23	8%	0.79

* χ^2 ^test

^† ^Self-reported chronic diseases, illnesses or disabilities diagnosed by a physician, dentist, or psychologist, produced symptoms or required treatment during the last 12 months

Regardless of frequent mentions of chronic conditions the majority of both male students (86%, n = 976) and female students (84%, n = 1 701) assessed their health status as 'good' or 'fairly good' in the 'Student Health Survey 2004' (Table 7).

**Table 7:** Self-reported health status by gender among Finnish undergraduate students in the 'Student Health Survey 2004'

	Men		Women	
	(n = 1 132)		(n = 2 021)	

Health Status				
Good	625	55%	923	46%
Fairly good	351	31%	780	39%
Average	130	11%	276	14%
Rather poor	21	2%	30	1%
Poor	1	0.1%	4	0.2%
Missing data	4	0.4%	8	0.4%

There was no statistical significance between the group differences of male or female non-users and users in self-reported health status (Table 8).

**Table 8:** Self-reported health status of Finnish female and male students in the 'Student Health Survey 2004' by use of the web-based health advice service and the statistical significance of the difference

	Men					Women				
	Non-users		Users		P*	Non-users		Users		P*

	(n = 1 019)		(n = 92)			(n = 1 720)		(n = 277)		

Health Status										
Good	566	56%	51	55%		798	46%	115	42%	
Fairly good	316	31%	30	33%		649	38%	125	45%	
Average	115	11%	10	11%		240	14%	35	13%	
Rather poor	21	2%	1	1%		28	2%	2	1%	
Poor	1	0.1%	0	0%	0.96	5	0.3%	0	0%	0.14

* χ^2 ^test

## Discussion

### Material and Methods; strengths and limitations

The general rate of response (63%) for the 'Student Health Survey 2004' was good compared with recent studies of the Finnish student population with response rates ranging from 44% to 48% [29,30]. The response rate among female students in the 'Student Health Survey 2004' was high (74%) and 2 001 out of 2 021 (99%) of female respondents answered target questions concerning the web-based health advice service, chronic conditions, and health status. The study results can thus be considered representative of the female student population at universities in Finland.

The response rate among male students in the 'Student Health Survey 2004' remained just under 50% (49%). The results concerning male students should thus be generalized cautiously. Low response rates in health surveys are not unusually observed in young male populations. In 2004 the response rate for a Finnish national health survey was lowest (54%) among young men aged 15 – 24 in an evaluation by sex and age group [31].

The main limitation of our study is insufficiency of result generalization at the population level. University students are not representative of the whole population. However, it is possible to regard these university students as 'pilot population' representing the young adult population of the information society.

The study design and statistical methods (cross-tabulation and χ^2 ^test) chosen for our study enable us to characterize the users of web-based health advice service in general. However, statistical methods used in our study are insufficient for indicating the dependence of variables on one another, or for showing the order of importance of variables. In addition, no corrections for multiplicity were applied as this is an explorative study. As the number of statistical tests is quite large in our study, p-values close to the significance limit of 0.05 should be interpreted with caution.

Our method for estimating one's own health status is a widely adopted method for the holistic evaluation of health and wellbeing [32]. It gives information about a survey respondent's health status in relation to his reference group [33]. Health experienced by individuals has been shown to be an independent variable associated with mortality and morbidity that is also among young people [34].

Surveys questions chosen for our study concerning chronic conditions have been used in a similar form in recent surveys on the national population level and among the student population [31,35,36]. With regard to data on chronic conditions and medical diagnosis, a survey based on data collection from patient records could have given more accurate data.

The percentage (12%) of university students who reported use of the web-based health advice service was not unexpected – it was remarkably lower than the percentage (75%) of students reporting face-to-face contact with the FSHS or the percentage of students (49%) reporting that they had visited the FSHS homepage [9]. However, there are no earlier study results that could be compared with our results to back up this view. Even if medical-based consultation services are popular they have still not been widely used as a study focus [18].

### Comparison with Prior Work

Mental health problems are increasing in western countries in general, as well as among Finnish university students [28,37,38]. Finnish national registers of medical consumption show a remarkable increase of anti-depressive medicine consumption during the last 4 years [39].

Mental health problems are of high clinical importance in adolescent medicine and school and student health care settings. Three out of four mental health problems among adults had their onset already before age of 24 [40]. Psychological distress has been shown to associate with frequent use of health care services, but only a minority of distressed young people has been seeking help by mental care professionals [41].

Thus, the association between the use of the web-based health service and psychiatric conditions is result of high clinical importance in this study. This result is supported by the results of earlier studies concerning the association between chronic conditions and the active general use of the Internet for health purposes [3,21,23,26]. It also states that the Internet as method and written form of expression is suitable for sensitive, stigmatized, or disquieting health issues [18,25,42]. An anonymous way of asking questions on delicate themes can provide the opportunity to test the capability of health service providers regarding sensitive issues. These aspects can be emphasized when it comes to mental health problems and can contribute to the patients' general motivation and courage to seek help in health care.

The prevalence of allergic disorders has increased during the last decade [30]. The prevalence of allergic rhinitis in the university student population has been 17–20% in recent studies [28,36,43]. Students with allergic rhinitis perceived their health as being worse than that of the control group; they had more symptoms and medication, and also used health services more frequently [44]. Finnish national registers of medicine consumption show an increase in systemic antihistamine consumption over recent years [39].

In our study male users of the web-based health advice service had been diagnosed more frequently with allergic rhinitis and conjunctivitis compared with male students not reporting use of the web-based health service. This finding can be explained as young a man's way of handling a chronic health problem using an eHealth service with easy access. The service can be a way of avoiding congested appointments, or phone services. Among male students the most requested eHealth services were appointment scheduling and prescription renewals [9]. Men who used anonymous Internet consultation stated that expressed interest in the explanation of symptoms was the main reason for using the service [18]. An association between a recently received chronic diagnosis and subscribing to an electronic messaging service was discovered in a recent study, where most of the patients were well-educated middle-aged men [45]. Our study results concerning the association of allergic disorders and the use of web-based health advice service supported by recent corresponding study findings indicate that focused eHealth services could provide support for managing chronic health problems [18,45].

Our study results concerning the association between cardiovascular diseases, and kidney and urinary tract disorders and the use of the web-based health advice service among women have only limited clinical value. The number of women suffering from these conditions was quite low.

The association between self-reported lower health status and use of the Internet for health problems is not supported by our results – self reported health status did not show any association with the use of the web-based health advice service among Finnish university students [3,22,46]. Our results can be explained by the primary recruitment of our study population; they are all young and well-educated and the incidence rates of chronic conditions, with multiple loading or daily-life limiting symptoms are low. In addition the use of a health service with easy access and low threshold by highly educated and relatively healthy young women can be explained by a general interest in and awareness of health issues. As far as young adults are concerned, web-based services are perhaps best suited to topics that are important in terms of health education regardless of the user's self-evaluated health status or diagnosed chronic illnesses. Typical topics of this type would include e.g. family planning.

## Conclusion

Our main results were: 1) In 2004 12% of the Finnish university student population reported use of a web-based health advice service, in which a student can ask health care professionals anonymously about matters related to health and illnesses. 2) The proportion of male students who reported allergic rhinitis or conjunctivitis was greater among the service users than non-users. 3) The proportion of female students reporting chronic mental health problems was greater among users than non-users. 4) Self-reported health status was not associated with the use of the web-based health advice service.

Our findings differ from recent study results concerning self-reported health status and use of Internet-based health services. Unlike several survey findings regarding adult populations in general our study indicates that among young, highly educated adults, the use of a web-based health advice service is not associated with self-reported health status. However there were specific chronic conditions that we could show as having an association with the use of an anonymous Internet consultation.

As a consequence of our results, new study questions can be recognized: Are the users of the web-based health advice service those who are also active users of traditional forms of health services? Are they seeking a second opinion associated with face-to-face visits? More research data is needed to evaluate the role of a web-based health advice service in supplementing the traditional forms of health services in order to discover the main benefits of this type of eHealth services.

## Competing interests

The author(s) declare that they have no competing interests.

## Authors' contributions

JC designed and coordinated the study, participated in the data analysis and drafted the manuscript. TH participated in the design of the study, performed the statistical analysis and helped to draft the manuscript. KK participated in the design of the study and helped to draft the manuscript. All authors read and approved the final manuscript.

## Pre-publication history

The pre-publication history for this paper can be accessed here:


